# Retraction: Enhancing radiosensitivity of melanoma cells through very high dose rate pulses released by a plasma focus device

**DOI:** 10.1371/journal.pone.0294549

**Published:** 2023-11-14

**Authors:** The PLOS ONE Editors

Following the publication of this article [1, corrected in 2], concerns were raised regarding Figs 2, 3, 4 and 9. Specifically,

- In Fig 2d, band 2 in the PFMA-3 p-P53 panel appears similar to band 1 in the XRT p-P53 panel. Additionally, band 1 in the XRT panel appears to be flanked by vertical discontinuities.
- In Fig 2d, band 6 in the PFMA-3 p-P53 panel appears to be flanked by horizontal and vertical discontinuities.
- In Fig 3 the following panels appear similar when levels are adjusted to visualise the background:
  ○ The 24 hour PFMA-3 panel in Fig 3a and the 24 hour XRT panel in Fig 3b.
  ○ The ctrl PFMA-3 panel in Fig 3a and the 48 hour PFMA-3 panel in Fig 3b.
  ○ The 48 hour PFMA-3 panel in Fig 3a and the 3 hour XRT panel in Fig 3c
- In Fig 3d, bands 1–4 in the XRT p-P53 panel appear similar.
- In Figure 4d, bands 2 and 5 in the XRT Cleaved Caspase-9 panel appear similar.
- In Fig 9a, when levels are adjusted to visualise the background in the ctrl BODIPY panel there is a vertical discontinuity on the left side of the panel.

The authors stated that underlying data for Figs 2, 3, and 4 were no longer available. The authors concurred with the assessment that band 2 and band 1 in the respective Fig 2d panels appeared similar. The PLOS Editors remain concerned about this figure. The authors stated the concerns in Fig 3a, 3b, and 3c were likely due to errors made during figure preparation. The authors stated that whilst similarities could be observed between bands denoted above in Fig 3d and Fig 4d, they asserted that these bands were not the same images. The authors stated the cause of the vertical discontinuity in Fig 9a was potentially caused by an error in the editing software used to process the image and provided the original underlying image to resolve the concern.

In light of the concerns affecting multiple figure panels that question the reliability of these data, the *PLOS ONE* Editors retract this article.

FB agreed with the retraction and apologized for the issues in the article. GCastellani, MS, LI, IZ, AMM, AC and EO did not agree with the retraction. SR, AT, DM and GCucchi either did not respond directly or could not be reached.
